# Knowledge-guided learning methods for integrative analysis of multi-omics data

**DOI:** 10.1016/j.csbj.2024.04.053

**Published:** 2024-04-30

**Authors:** Wenrui Li, Jenna Ballard, Yize Zhao, Qi Long

**Affiliations:** a Department of Biostatistics, Epidemiology and Informatics, Perelman School of Medicine, University of Pennsylvania, 423 Guardian Drive, Philadelphia, 19104, PA, USA; b Graduate Group in Genomics and Computational Biology, Perelman School of Medicine, University of Pennsylvania, 3700 Hamilton Walk, Philadelphia, 19104, PA, USA; c Department of Biostatistics, School of Public Health, Yale University, 60 College Street, New Haven, 06510, CT, USA

**Keywords:** Knowledge-guided learning, Multi-omics, Integration, Prediction, Feature selection, Clustering, Dimension reduction

## Abstract

Integrative analysis of multi-omics data has the potential to yield valuable and comprehensive insights into the molecular mechanisms underlying complex diseases such as cancer and Alzheimer's disease. However, a number of analytical challenges complicate multi-omics data integration. For instance, -omics data are usually high-dimensional, and sample sizes in multi-omics studies tend to be modest. Furthermore, when genes in an important pathway have relatively weak signal, it can be difficult to detect them individually. There is a growing body of literature on knowledge-guided learning methods that can address these challenges by incorporating biological knowledge such as functional genomics and functional proteomics into multi-omics data analysis. These methods have been shown to outperform their counterparts that do not utilize biological knowledge in tasks including prediction, feature selection, clustering, and dimension reduction. In this review, we survey recently developed methods and applications of knowledge-guided multi-omics data integration methods and discuss future research directions.

## 1 Introduction

Rapid advances in technologies have led to collection of various types of -omics data, such as genomics and proteomics data, in many biomedical studies. One notable example is the Alzheimer's disease neuroimaging initiative (ADNI) study [35], which collected multi-omics data from Alzheimer's disease (AD) patients, mild cognitive impairment subjects, and elderly controls. See Table 1 for several representative multi-omics databases. Integrative analyses of these datasets have the potential to deliver more comprehensive insights into the biological systems under study than is possible with individual modalities. For example, an integrative multi-omics approach revealed novel molecular and pathway alterations in Alzheimer's disease and led to better prediction of cognitive decline [9]. At the same time, there are a number of analytical challenges in integrative analysis of multi-omics data. For instance, -omics data are usually high-dimensional, leading to the classical small *n* large *p* problem. In addition, when individual genes in important pathways have relatively weak signal, it can be difficult to detect them on their own. To address these challenges, there is a growing body of literature on knowledge-guided learning methods for integrative analysis of multi-omics data that can incorporate biological knowledge such as functional genomics and functional proteomics via graph representations.

**Table 1:** List of multi-omics databases.

Database Name	Modalities	Biological Domain	Source
**TCGA** (The Cancer Genome Atlas) [42]	Genomics, epigenomics, transcriptomics, proteomics	Cancer	https://www.cancer.gov/ccg/research/genome-sequencing/tcga
**CCLE** (Cancer Cell Line Encyclopedia) [6]	Genomics, transcriptomics, epigenomics, proteomics, metabolomics	Cancer	https://sites.broadinstitute.org/ccle/
**CPTAC** (Clinical Proteomic Tumor Analysis Consortium) [12]	Genomics, proteomics (proteogenomics), imaging	Cancer	https://proteomics.cancer.gov/programs/cptac
**METABRIC** (Molecular Taxonomy of Breast cancer International Consortium) [11]	Genomics, transcriptomics, clinical	Breast Cancer	https://www.mercuriolab.umassmed.edu/metabric
**TARGET** (Therapeutically Applicable Research to Generate Effective Treatments) [1]	Genomics, transcriptomics, epigenomics	Pediatric cancers	https://www.cancer.gov/ccg/research/genome-sequencing/target/using-target-data
**ADNI** (Alzheimer's Disease Neuroimaging Initiative) [35]	Imaging, genetics, clinical, biospecimen	Alzheimer's disease	https://adni.loni.usc.edu/
**The Aging Atlas Database**[2]	Genomics, transcriptomics, epigenomics, proteomics, pharmacogenomics, metabolomics	Age-related changes	https://ngdc.cncb.ac.cn/aging/index
**MVIP** (Multi-omics Portal of Virus Infection) [39]	Genomics, transcriptomics, epigenomics	Virology	https://mvip.whu.edu.cn/
**iMETHYL**[20]	Genomics, transcriptomics, epigenomics (DNA methylation)	Genetics, biology, molecular biology	http://imethyl.iwate-megabank.org/

Extensive research has yielded much information on the association structure among features (e.g., genes and proteins) and underlying networks that can be represented by graphs. For instance, the Kyoto Encyclopedia of Genes and Genomes (KEGG) project [17] links genomic information with higher order functional information by representing higher order functions as a network of interacting molecules. Another example is the Search Tool for the Retrieval of Interacting Genes/Proteins (STRING) [29], which systematically collects and integrates protein–protein physical interactions and functional associations. See Table 2 for several notable databases containing various types of biological knowledge.

**Table 2:** Representative databases for biological knowledge.

Database Name	Biological Knowledge	Source
**KEGG** (Kyoto Encyclopedia of Genes and Genomes) [17]	Molecular interaction, reaction and relation networks	https://www.genome.jp/kegg/pathway.html
**Reactome** (Reactome Pathway Database) [10]	Signaling and metabolic pathways	https://reactome.org/
**GTEx** (Genotype-Tissue Expression) [27]	Tissue-specific gene expression	https://gtexportal.org/home/
**STRING** (Search Tool for the Retrieval of Interacting Genes/Proteins) [29]	Protein-protein interaction networks	https://string-db.org/
**PathBank**[43]	Metabolic, signaling, disease, drug, and physiological pathways	https://www.pathbank.org/
**Pathway Commons**[8]	Biological pathway and interactions: biochemical reactions; gene regulatory networks; protein, nucleic acid, small molecule interactions	https://www.pathwaycommons.org/
**BioCyc**[18]	Metabolic pathways, regulatory networks	https://biocyc.org/
**WikiPathways**[36]	Signaling pathways	https://www.wikipathways.org/
**GRNdb**[13]	Gene regulatory networks among transcription factors and genes	http://www.grndb.com/
**miRTarBase**[16]	miRNA-target interactions	https://mirtarbase.cuhk.edu.cn/~miRTarBase/miRTarBase_2022/php/index.php
**GRAND**[7]	Gene regulatory networks among transcription factors, miRNAs and genes across biological states	https://grand.networkmedicine.org/
**RegNetwork**[26]	Gene regulatory networks among transcription factors, miRNAs and genes	https://regnetworkweb.org/
**BioGRID** (Biological General Repository for Interaction Datasets) [33]	Protein and genetic interactions	https://thebiogrid.org/

**Fig. 1:**
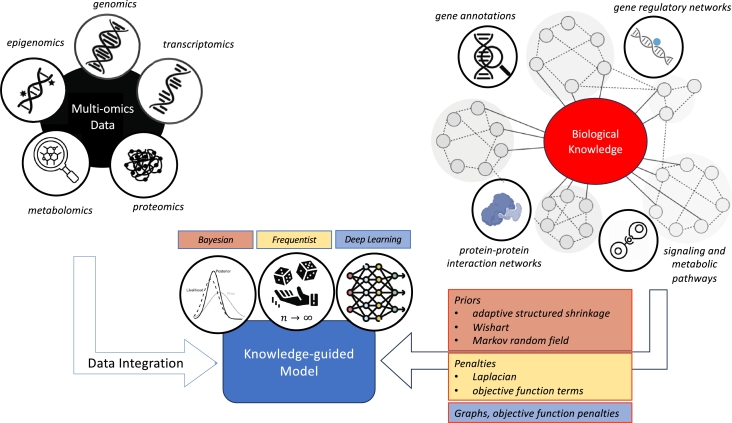
Knowledge-guided multi-omics data integration methodology.

**Fig. 2:**
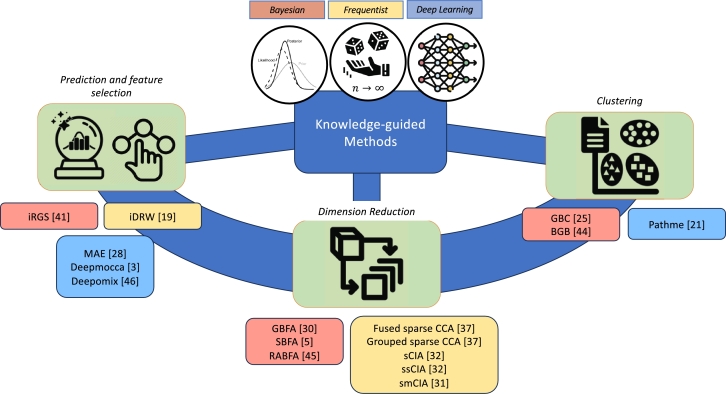
Overview of knowledge-guided multi-omics data integration methods. The methods are grouped based on their applications and are color coded as per their approaches.

Recent methodological research has provided strong evidence that knowledge-guided learning methods for integrative analysis of multi-omics data outperform their counterparts that do not use graph information on both supervised learning and unsupervised learning tasks. The results from knowledge-guided learning methods are more biologically meaningful and interpretable, and provide insights into the molecular mechanisms underpinning complex diseases such as cancer and AD. Knowledge-guided supervised learning methods can be used to construct prediction models for disease risk and progression, and identify important features that are highly associated with clinical end points. Knowledge-guided unsupervised learning methods can be used to identify disease subtypes and perform dimensionality reduction. The approaches utilized by the methods in this review to incorporate prior biological knowledge can be classified into three categories: Bayesian, frequentist, and deep learning. The Bayesian approach permits the use of biological knowledge by specifying prior distributions and producing posterior distributions for parameters in the model. The frequentist approach incorporates biological knowledge through penalty functions and yields point estimates for parameters. Deep learning approaches utilize prior biological knowledge by including graphs constructed based on the biological information (e.g. graph convolutional neural network) or imposing network constraints (e.g. autoencoder). Fig. 1 summaries the key components in the three approaches. Additionally, the applications of these methods can be grouped into three categories: prediction and feature selection, clustering, and dimension reduction. Fig. 2 provides a schematic representation of the knowledge-guided multi-omics data integration methods grouped according to their applications.

In this review, we survey the recent advances in knowledge-guided learning methods for integrative analysis of multi-omics data. We organize our presentation by application: prediction and feature selection, clustering, and dimension reduction. Note that we review knowledge-guided statistical and deep learning methods for multi-omics data integration published since 2018, while Zhao et al. [47] focuses on knowledge-guided statistical methods for analysis of single-omics data published by 2019.

## 2 Prediction and feature selection

Several knowledge-guided learning methods have been proposed to build prediction rules for clinical outcomes and identify important features that are highly associated with clinical end points. The outcomes include, but are not limited to, disease status, progression-free interval, survival time, and prognosis.

Wang et al. [41] developed a Bayesian framework that integrates multi-omics data and biological knowledge to infer risk genes driving genome-wide association studies (GWAS) signals. Their method probabilistically ranked genes at each GWAS locus based on supporting evidence from multi-omics data and correlation among the genes. The correlation of genes was implicitly derived from the gene network, with the rationale that disease genes are more densely connected and are therefore more highly correlated. Their method was applied to schizophrenia GWAS data. A generic gene–gene network was constructed from Gene Ontology [4]. They identified a set of high-confidence risk genes that are significantly enriched for heritability and are also enriched in targets of approved drugs. Later, Kim et al. [19] proposed an integrative directed random walk-based method utilizing biological knowledge for more effective feature selection and prediction. They designed a directed gene-gene graph for gene expression and copy number data using the biological information from the KEGG database [17]. Then, the integrative directed random walk was applied to the gene-gene graph. When applied to multiple genomic profiles for breast cancer and neuroblastoma, their method identified biologically significant pathways and genes that are highly correlated with cancer and had superior survival prediction performance compared to several state-of-the-art methods.

More recently, Ma and Zhang [28] proposed a multi-view autoencoder model with network constraints that can simultaneously integrate multi-omics data and biological knowledge. The biological knowledge was incorporated into the model as inductive biases to increase model generalizability. They employed a graph Laplacian regularizer to encode the biological network into the model architecture. The regularizer reduces the inconsistency between the learned feature representation and the biological network. They applied the model to bladder urothelial carcinoma and brain lower grade glioma datasets as well as The Cancer Genome Atlas (TCGA) pan-cancer dataset. These datasets contain gene expression, miRNA expression, protein expression, and DNA methylation and clinical data. The biological network information was obtained from STRING [29]. Their method outperformed traditional methods and conventional deep learning models on predicting clinical outcomes (e.g., progression-free interval and overall survival) from multi-omics data. Later, Althubaiti et al. [3] developed a computational model using graph convolutional neural networks built upon a graph constructed from prior knowledge of the functional interactions between genes and their products. In the graph, nodes represent genes, transcripts, and proteins, and edges between nodes represent functional interactions between them. They designed a set of mapping functions to map the information from the multi-omics data to nodes in this graph. A graph convolutional neural network combined with Cox regression was then used to predict patient survival time. They applied the model to multi-omics cancer data from TCGA [42]. The biological information was obtained STRING [29]. Their method predicted survival time for individual patient samples and outperformed most existing survival prediction methods. They also identified genes that have been demonstrated to be closely related to cancer survival. Zhao et al. [46] proposed a scalable and interpretable multi-omics feed-forward neural network framework that enables the non-linear combination of variables from different omics datasets and incorporates prior biological information. They used multi-omics data at the gene-level as the input data to the gene layer. The gene layer nodes were connected with a functional module layer according to the prior biological information. Each node in the functional module layer was a non-linear function of the values at the different molecular levels of the genes it contained. By incorporating prior biological knowledge, their model could extract significant modules to understand the underlying mechanisms for diseases. Their method was applied to multi-omics and survival time data from TCGA [42]. The biological information was obtained from KEGG [17] and Reactome [10]. Benchmark experiments showed that their framework outperforms other cutting-edge methods for integrating multi-omics data and predicting the survival time. In the case study of lower grade glioma, they identified functional pathways associated with prognosis groups that have been confirmed by previous studies. Thus, knowledge-guided deep learning methods can identify complex non-linear patterns in data while also being efficient for processing large volumes of multi-omics data.

## 3 Clustering

The goal of clustering is to group patients based on their similarities. It has been widely used to uncover disease subtypes, which is important for developing tailored medicine and providing more precise treatment for individual patients. There are a few knowledge-guided learning methods that incorporate biological knowledge in clustering to improve subtyping accuracy and yield more biologically interpretable results. Li et al. [25] proposed a generalized Bayesian biclustering approach to jointly analyze multi-omics data while incorporating biological information. Their method can handle multiple data types, for example, binomial data such as single nucleotide polymorphism (SNP) data or negative binomial data such as RNA sequencing data. To incorporate biological knowledge, they employed a Bayesian adaptive structured shrinkage prior on the factor loading matrix. The prior encourages one variable to load on a factor if another connected variable has a non-zero loading on the same factor. For example, if two genes are connected in a pathway, they are encouraged to be selected (or not) simultaneously within a bicluster. Therefore, the selected feature set in each bicluster tends to include gene pathways rather than individual genes, resulting in biologically more meaningful results. They conducted biclustering analysis using microarray gene expression data, DNA methylation data, and DNA copy number data from a TCGA study in glioblastoma multiforme. The biological information was obtained from the KEGG database [17]. The higher correlation between subgroups identified by their method and patient survival time compared to other biclustering methods suggested that the clusters detected by their method are more clinically meaningful. Later, Zhang et al. [44] proposed a novel constrained Wishart prior to incorporate biological knowledge in biclustering analysis. The prior encourages the simultaneous selection or non-selection of connected features within a bicluster. In addition, their method effectively addresses the diagonal-dominant issue of the graph-incorporated prior in Li et al. [25], and can handle scenarios involving larger network sizes or constructed networks containing noise. Simulation studies showed that their method outperformed existing biclustering methods including Li et al. [25]. When they conducted biclustering analysis using SNPs and gene expression data from ADNI, their method yielded the best clustering performance. Furthermore, all enriched pathways identified by their method had previously been demonstrated to be closely related to AD.

In addition, Lemsara et al. [21] proposed a multi-modal sparse denoising autoencoder framework that allows for the incorporation of biological knowledge to cluster patients. They mapped the multi-omics features to pathways and estimated a per-patient score for each pathway via multi-modal sparse denoising autoencoders. Then, they combined scores of multiple pathways into a profile for each patient, which could then be used to cluster patients. They applied the framework to cluster patients in several cancer datasets from TCGA [42] using gene expression, miRNA expression, DNA methylation and copy number variation. The biological information was obtained from Nature Pathway Interaction Database [38]. Their method identified biologically plausible disease subtypes and showed competitive clustering performance compared with several competing methods.

## 4 Dimension reduction

Projecting high-dimensional multi-omics features into a low dimensional space allows for better understanding and visualization of the structure of the data and helps to assess relationships among multiple data sets. Several knowledge-guided learning methods have been proposed for dimensionality reduction in multi-omics data.

Factor analysis is a popular tool for modeling individual and shared structures in multi-omics data. It infers a lower number of unobserved variables called latent factors that capture the majority of the variation in the original high-dimensional data. Min et al. [30] developed a generalized Bayesian factor analysis framework that can jointly analyze multi-omics data and incorporate biological information. They employed the spike and slab lasso prior to impose sparsity on the factor loadings and the Markov random field prior to incorporate network information. The priors encourage the connected variables to share common factors. They applied the model to transcript profile data, mRNA expression data and proteomics profiling data from NCI-60 cell lines. The biological information was obtained from the KEGG database [17]. The application results showed that their method could deliver more biologically meaningful outcomes than methods that do not incorporate graph information. Later, Bao et al. [5] proposed a hierarchical structural Bayesian factor analysis model that successfully incorporates prior biological information without suffering the phase transition problem in Min et al. [30]. To incorporate biological knowledge, they employed a Bayesian adaptive structured shrinkage prior on the factor loading matrix. The prior encourages connected variables to share common factors. In addition, their method can handle both continuous data (e.g. gene expression data) and discrete data (e.g. SNPs) simultaneously. They used the latent factors learned through integrative analysis of the genotyping, gene expression, brain regional level amyloid deposition data from the ADNI database to predict cognitive score. The gene–gene interaction network and brain functional network were obtained from Greene et al. [15] and Glasser et al. [14], respectively. Their method achieved the best prediction accuracy compared with the other state-of-the-art factor-analysis-based methods including Min et al. [30]. More recently, Zhang et al. [45] proposed a novel constrained Wishart prior to incorporate the biological graph knowledge in factor analysis. Their method effectively addresses the diagonal-dominant issue of the graph-incorporated prior in Bao et al. [5], and it is robust to noisy edges that are inconsistent with the structure of the factor loadings. Their method encourages connected variables to share common factors. They applied the factor model to microarray gene expression data, DNA methylation data and DNA copy number data from a TCGA study in glioblastoma multiforme. The biological information was obtained from the KEGG database [17]. Their method outperformed the existing competitors including Min et al. [30] when the learned factors were used in survival analysis, and it detected genes that play important roles in the different subtypes of glioblastoma multiforme.

Canonical correlation analysis and coinertia analysis are multivariate statistical methods frequently used in integrative analysis and have become popular in analysis of multi-omics data. Safo et al. [37] developed statistical methods for sparse canonical correlation analysis that incorporate biological information. To do this, they extended and investigated two types of network-based penalties: the grouped penalty by Pan et al. [34] and the fused lasso penalty by Tibshirani et al. [40]. Their method utilizes biological information such as gene and metabolomic networks to guide selection of important metabolites and transcripts. They conducted integrative analysis of the transcriptomic and metabolomic data from the Predictive Health Institute study. The gene and metabolomic network information was obtained from KEGG [17] and mummichog [23], respectively. Their method identified a number of gene and metabolic pathways that are known to be associated with cardiovascular diseases. As with canonical correlation analysis, biological knowledge has also been incorporated into coinertia analysis. Min et al. [32] proposed a coinertia analysis method that incorporates biological knowledge to assess dependence between two -omics data sets. To incorporate biological knowledge, they adopted the Laplacian penalty function proposed by Li and Li [22]. The penalty function encourages connected variables to be selected or not selected together. Simulation studies demonstrated that their method achieved the best or close to the best performance compared to the existing co-inertia analysis methods. Their method was applied to the integrative analysis of gene expression and protein abundance data from NCI-60 cancer cell lines. The biological information was obtained from the KEGG database [17]. Their method identified biologically meaningful genes and proteins for cancer. Later, Min and Long [31] extended the framework in Min et al. [32] to multiple co-inertia analysis, which can assess relationships and trends in multiple datasets. They employed the Laplacian penalty to incorporate biological information. The connected variables were encouraged to be selected or excluded at the same time. Their method was applied to two gene expression datasets and one protein abundance dataset from NCI-60 cell line data. The biological information was obtained from KEGG database [17]. They projected the high-dimensional -omics features to a lower dimensional space and identified a subset of biomarkers that are suggested in the literature to be related with cancer disease.

## 5 Conclusion

The knowledge-guided approach for data integration is a powerful strategy to analysis of multi-omics data in modern biomedical research. This paper reviews some of the recent developments in this space. Although knowledge-guided methods have been shown to yield more biologically meaningful and interpretable results than those that do not use graph information, there is still ample room for methodological development and improvement. One future direction is to account for the noise in the biological knowledge represented by a graph. The graphs extracted from existing databases or relying on subject matter expertise are known to be incomplete and may contain false edges. To our best knowledge, existing knowledge-guided methods have largely ignored the important issue of network misspecification and routinely use the given network directly in their models without accounting for noise. We note that while the recent work by [48] addressed a related problem for handling missing edges in only part of the graph via a multiple-imputation approach, their method is limited in scope and cannot be directly applied to diverse and general settings involving noisy networks with varying degrees of misspecification. Li et al. [24] used a latent scale model to account for network noise in graph-guided Bayesian modeling of structured data, which could potentially be extended to integrative analysis of multi-omics data. Another area for future research is to develop computationally efficient algorithms. Most of the existing knowledge-guided Bayesian methods may not be scalable to analysis of ultra-high-dimensional -omics data that may include hundreds of thousands or even millions of features.

## CRediT authorship contribution statement

**Wenrui Li:** Conceptualization, Data curation, Investigation, Methodology, Supervision, Visualization, Writing – original draft. **Jenna Ballard:** Data curation, Investigation, Validation, Writing – original draft. **Yize Zhao:** Writing – review & editing. **Qi Long:** Conceptualization, Funding acquisition, Methodology, Project administration, Resources, Supervision, Writing – review & editing.

## Declaration of Competing Interest

The authors declare that they have no known competing financial interests or personal relationships that could have appeared to influence the work reported in this paper.
